# Cytotoxic Effects of Plant Secondary Metabolites and Naturally Occurring Bioactive Peptides on Breast Cancer Model Systems: Molecular Mechanisms

**DOI:** 10.3390/molecules29225275

**Published:** 2024-11-07

**Authors:** Diana Zasheva, Petko Mladenov, Silvina Zapryanova, Zlatina Gospodinova, Mariyana Georgieva, Irina Alexandar, Valentin Velinov, Dimitar Djilianov, Daniela Moyankova, Lyudmila Simova-Stoilova

**Affiliations:** 1 Institute of Biology and Immunology of Reproduction, Bulgarian Academy of Sciences, Tsarigradsko Shosse, 73, 1113 Sofia, Bulgaria; zasheva.diana@yahoo.com (D.Z.); silvina_z@abv.bg (S.Z.); 2 Agrobioinstitute, Agricultural Academy, bul. “Dragan Tsankov” 8, 1164 Sofia, Bulgaria; mladenovpetko@yahoo.com (P.M.); d_djilianov@abi.bg (D.D.); dmoyankova@abi.bg (D.M.); 3 Institute of Plant Physiology and Genetics, Bulgarian Academy of Science, “Acad. Georgi Bonchev” Str., Bl. 21, 1113 Sofia, Bulgaria; zlatina.go@abv.bg (Z.G.); stamova@bio21.bas.bg (M.G.); valentin.velinov82@gmail.com (V.V.); 4 Institute of Molecular Biology “Rumen Tzanev”, Bulgarian Academy of Sciences, “Acad. Georgi Bonchev” Str., Bl. 21, 1113 Sofia, Bulgaria; ialexandar@yahoo.com

**Keywords:** breast cancer, MCF7, MDA-MB231, cytotoxicity, secondary metabolites, bioactive peptides

## Abstract

Breast cancer is the second leading cause of death among women, and the number of mortal cases in diagnosed patients is constantly increasing. The search for new plant compounds with antitumor effects is very important because of the side effects of conventional therapy and the development of drug resistance in cancer cells. The use of plant substances in medicine has been well known for centuries, but the exact mechanism of their action is far from being elucidated. The molecular mechanisms of cytotoxicity exerted by secondary metabolites and bioactive peptides of plant origin on breast cancer cell lines are the subject of this review.

## 1. Introduction

The problem of effective cancer therapy is becoming increasingly important. The number of patients diagnosed with a tumor and the number of mortal cases among those patients have been expanding over the past few years. In 2018 alone, the number of dead cancer patients was about 10 million, as reported by the World Health Organization [1]. Concerning breast cancer, 1.38 million women were diagnosed in 2012 [2], and by 2020 this number had increased to 2.3 million [3]. Breast cancer is the most common cancer among women worldwide by 2021, although about 1% of breast cancer patients are men [4]. Breast cancer cases account for 12% of all cancer cases and can be found in teenage girls, in women of reproductive age, and in menopausal women with preliminarily developed climax changes [5].

Commonly used methods of breast cancer treatment are surgery, chemotherapy, and radiation therapy. Surgical methods are invasive with a long recovery period and usually affect a woman’s quality of life. The surgical method is followed by chemotherapy and radiotherapy or a combination of them. Chemotherapeutic anticancer drugs are usually synthetic drugs and have cytotoxic effects. They are classified into several groups depending on the mechanism of action on cells. The following groups are used in anticancer therapy: microtubule-interacting agents (vincristine, taxol) [6], drugs with a topoisomerase-inhibiting effect (doxorubicin, podophyllotoxin) [7], alkylating agents such as melphalan, methotrexate, blocking cell metabolism [8], and DNA interacting agents such as docetaxel [9]. The molecular mechanisms of commonly used antitumor drugs are not very specific; they kill not only cancer cells but their targets could also be normal cells. This fact explains many side effects of chemotherapeutics. The same is the case with radiotherapy. These conventional therapeutic methods are associated with loss of normal blood cells, loss of body mass, and loss of hair; some of them are associated with vomiting and dizziness [2].

Long-term use of drugs in anticancer therapy can lead to the emergence of resistance to them in cancer cells (multidrug resistance, MDR) [10]. The MDR of cancer cells results from molecular mechanisms related to the inability of administered drugs to penetrate into the cells and trigger a mechanism of cancer cell elimination. These facts make drugs ineffective, and cancer development continues progressively. The probability of the emergence of multidrug resistance in cancer patients, together with the strong impact of the side effects of chemotherapy drugs on the treated organism, makes the search for new anticancer therapeutics so necessary and significant. Possible substances that could be expected to have little or no side effects are certain substances of plant origin. Plants have been used in medicine since ancient times. Generally, Chinese traditional medicine is based on them; Ayurveda in India is based on therapeutic methods using plants and substances of plant origin. Intensive studies of plant secondary metabolites and bioactive peptides are being carried out on cancer cell lines, a model system that enables the testing of plant derivatives on cell lines with characteristics of different types of breast cancer. The mechanisms of their cytotoxicity and their targets can be established at the cellular and molecular levels. The subject of this review will be studies of well-characterized substances among secondary metabolites and polypeptides of plant origin with cytotoxic effects on breast cancer cell lines with different characteristics and the molecular mechanisms mediating the reduction in cancer cell number as a result of plant substances application. Breast cancer cell lines are well-characterized and convenient model systems with the same cell signaling mechanisms as the primary breast tumors. The possible new search for metabolites with specific targets, depending on cell-line characteristics and cancer characteristics, will be discussed. The discovery of plant polypeptides targeting specific proteases with a high impact on cancer cell proliferation, invasion metastasis formation, and angiogenesis will be elucidated. Future prospects of searching for new plant metabolites and polypeptides with breast anticancer effects will be suggested.

## 2. Breast Cancer Types and Signal Pathways Related to the Breast Cancer Development

Depending on cancer origin, the types of breast cancer are ductal (85% of breast cancer cases; lobular, originating from the lobes (9–14%), and very aggressive atypical inflammatory forms (1–6% of the cases) [11]. The characteristics of the transformed cells are also very important. Depending on the expression of cell receptors—estrogen receptor (ER), progesterone receptor (PR), and human epidermal growth factor receptor 2 (HER2), breast cancer types are divided into four molecular subtypes: basal (triple negative, which does not express ER, PR, and HER2 (11 % of BC cases with an aggressive phenotype and lack of response to hormone replacement therapy), luminal A (ER+/PR+/HER2−, 60% of BC cases), luminal B (ER+/PR+/HER2+, 15% of BC cases), and HER2 overexpressing (ER-/PR−/HER2+, 14% of BC cases). The cellular transformation of breast cancer is characterized by changes in signaling pathways as a result of some risk factors such as genetic and environmental that could be predisposed to it, followed by changes in molecular mechanisms accompanied by mutations in different groups of genes: tumor-suppressor genes (*p53*, *NF1*, *NF2*), DNA repair system genes (*PTEN*, *BRCA1* and *BRCA2* genes), oncogenes (*RAS*, *MYC*, *Bcl2*, *RAF*), and genes involved in cell growth and metabolism [12]. These mutations lead to the acquisition of uncontrollable division of cancer cells, and invasion of the tissues where they are located and, therefore, can form blood vessels and metastasize.

**Figure 1:**
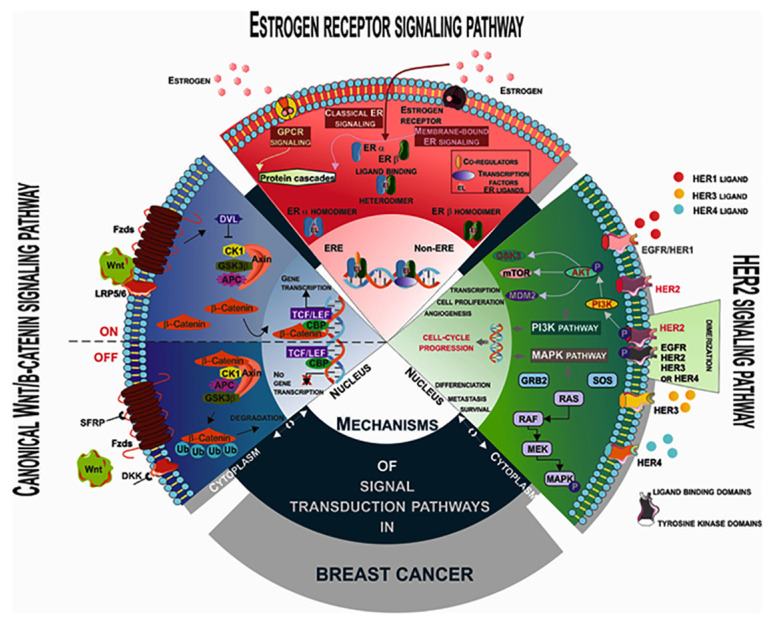
Main signaling pathways associated with breast cancer transformation.

The main signaling pathways associated with breast cancer transformation and progression (Figure 1) depend on the expression of the estrogen receptor and its associated signaling—ER alpha (ERα—a membrane receptor) and ER beta (ERβ—a nuclear receptor) [13,14,15,16]. All of them are linked with the processes of cell development directed by changes in cell cycle control and proliferation, cell differentiation and cell death, and cell migration and motility [17]. The pathway associated with cell proliferation is mediated by ERα interaction with cyclin D. Cyclin D is an activator of cyclin-dependent kinases 4 and 6, which mediate cell cycle progression from G1 to S phase [17,18]. This type of cancer responds to hormone replacement therapy, and estrogen-dependent cell line models such as MCF7 are used in studies to search for targeted therapeutic blocking estrogen receptor α in high levels and estrogen receptor β in low levels, whose homodimers are located in the cell cytoplasm, and their dimerization is needed for activation and nuclear translocation. They direct the transcription of different genes in the nucleus, or they are cofactors of various other transcription factors [18,19,20,21].

The HER2 receptor signaling pathway characterizes the breast cancer cell overexpressing the HER2 receptor (human epidermal growth factor receptor), which is a member of the endothelial growth factor receptor family of four receptors. They are receptor tyrosine kinases with extracellular, membrane-binding, and intracellular domains [22,23,24,25]. The HER2 receptor is a molecule that forms dimers with other molecules of the HER receptor families, and the binding of membrane ligands to the dimers enables phosphorylation of its intracellular domains [26,27,28]. This activates kinase signaling pathways such as mitogen-activated protein kinase (MAPK) and phosphatidylinositol 4,5-bisphosphate 3-kinase (PI3K) associated with breast tumorigenesis through accelerated proliferation and cell cycle progression [29,30,31,32]. High expression of HER2 in breast cancer cells increases the metastatic potential of breast tumors [30].

The third signaling pathway is the canonical Wnt/β catenin, whose deregulation has a strong impact on cell transformation, development, and progression of breast cancer. Wnt proteins are a family of glycosylated secreted proteins with a high impact on embryonal tissue development and mammary gland development. The interaction of Wnt proteins with their receptors leads to the maturation of Axin and disheveled proteins to the cell membrane. The inhibition of glycogen synthase kinase 3β protein is initiated, and its function as a Wnt protein inhibitor is blocked. The β-catenin ceases to be degraded in the proteasome and accumulates in the cytoplasm as a result of inhibition of the glycogen synthase kinase 3β protein, and its translocation into the nucleus is enabled. In the nucleus, it becomes a cotranslational activator of different oncogenes *MYC*, *CCND1* together with their translational activator CREB binding protein and T cell factor/lymphoid enhancer factor transcription factors [33,34,35,36,37,38]. The Wnt pathway is constitutively activated by an autocrine mechanism in breast cancer. Mutations in Wnt proteins have not been found in the genome of breast cancer cells, but the positive regulator of their signaling Dvl is amplified in 50% of breast cancers, and the Wnt-related inhibitor Frizzled protein 1 is lost in 78% of malignant cancers diseases of the breast, which correlates with poor prognosis development of cancer [39,40]. Wnt/β catenin signaling is activated in basal-like breast cancers [41,42], which is associated with poorer prognosis. The Wnt//β catenin signaling pathway drives tumor progression in HER2-positive breast cancers, which has been established by in vivo studies and plays a role in pathways involved in multiple resistance to standard antibreast cancer drugs [43].

Many other signaling pathways that have a role in normal breast development play an important role in breast cancer development if dysregulated, such as the cyclin-dependent kinase pathway that involves cell cycle progression. It is regulated by cyclins, cyclin-dependent kinases and their inhibitors [44]. Usually, breast cancer development is associated with the amplification of cyclin D1 and its high expression, respectively, the high expression of cyclin E in 60% of breast tumors and a mitogenic effect of cyclin D1 exerted by an estrogen-dependent mechanism. Decreased expression of the CDKI p27Kip1 has been established in breast cancers [45].

The Notch signaling pathway consists of five Notch ligands, which are transmembrane proteins named Delta-like 1,3,4 and Jagged (Jag) 1,2. Notch signaling is directed by proteolytic reactions activated by ligand-receptor binding. The result of proteolysis is the formation of a Notch intracellular domain transcription factor that regulates downstream target genes. Higher expression of Notch signaling genes leads to poor cancer prognosis [46,47]. Breast cancer etiology is linked to sonic hedgehog (SHH) signaling with a role in mouse glandular development. Disruptions of two of the SHH pathway-related factors, patched homolog-1 (PTCH-1) or glioma-associated oncogene-2 (GLI-2), are observed in breast cancer [47,48]. The breast tumor kinase (BTK) signaling pathway is another signaling pathway with a role in breast cancer. It is a nonreceptor tyrosine kinase overexpressed in more than 60% of breast cancer cases, and EGFR-regulated signaling is impaired in these cancers, which significantly increases MAPK activity and thus leads to fast proliferation and migration of breast cancer cells [49]. The PI3K/AKT/mTOR signaling pathway is another one with an impact on breast cancer development; more PI3K mutations are features of breast cancer predisposition. They lead to cellular dedifferentiation of basal and luminal progenitor cells, rendering them of multilineage potential [50]. Akt kinase and downstream mTOR hyperactivation leads to resistance to endocrine therapy [51]. Inhibition of mTOR is a good candidate for targeted therapy due to the possibility of restoring the antiestrogen sensitivity of cancer cells [51]. The knowledge of the molecular mechanisms of breast cancer transformation provides an opportunity to search for new targeted therapeutic drugs that are more effective and less invasive. The interest in plant-derived compounds with anticancer activity has been revived in recent years and intense research on the molecular mechanisms of their action, especially targeting cancer signaling pathways, is being performed. Cancers, and especially breast cancer, are diseases related to different changes in the genome, oxidative stress, and a change in immune functions; the search for molecules which modulate the key elements associated with breast cancer transformation is a challenge in the studies related to breast cancer therapy.

## 3. Plant Secondary Metabolites with Anticancer Effects on Breast Cancer Cell Lines—Groups of Plant Secondary Metabolites and Molecular Mechanisms Mediating Their Cytotoxic Effects

Plant secondary metabolites are organic chemical substances that have an essential role in plant adaptation to changes in environmental factors and for their viability under stressful conditions. They have a protective role in plants against various stresses—abiotic, biotic, and oxidative ones. These metabolites are extractable from plants using organic solvents such as ethanol, methanol, and ethyl acetate [52]. Fractionation of the extracts on HPLC and LC results in fractions with a mixture of secondary metabolites. These fractions can be refractionated by a secondary fractionation, and their components can be characterized by nuclear magnetic resonance (NMR) and mass spectrometric analysis. These methods enable the use of an integrative multi-omics approach for the prediction of the potential of a plant metabolite to exert anticancer effects on tumor cells. Plant secondary metabolites are divided into the following groups: bioflavonoids, unsaturated fatty acids, tannins, alkaloids, and terpenoids [53]. Components with cytotoxic effects are usually searched among these groups of plant secondary metabolites, although they are exceptionally divergent with different structures, depending on the extracted plants and organs, their areal and living conditions. The anticancer effects of the studied secondary metabolites could be categorized into the following mechanisms. They usually block the cell cycle or initiate some of the cell death pathways (necrosis, apoptosis). They can mediate mechanisms related to the suppression of essential enzymes for cell proliferation or be of a structure that can block receptors with an essential role in the development, proliferation, and metastatic potential of cancer.

### 3.1. Flavonoids

**Figure 2:**
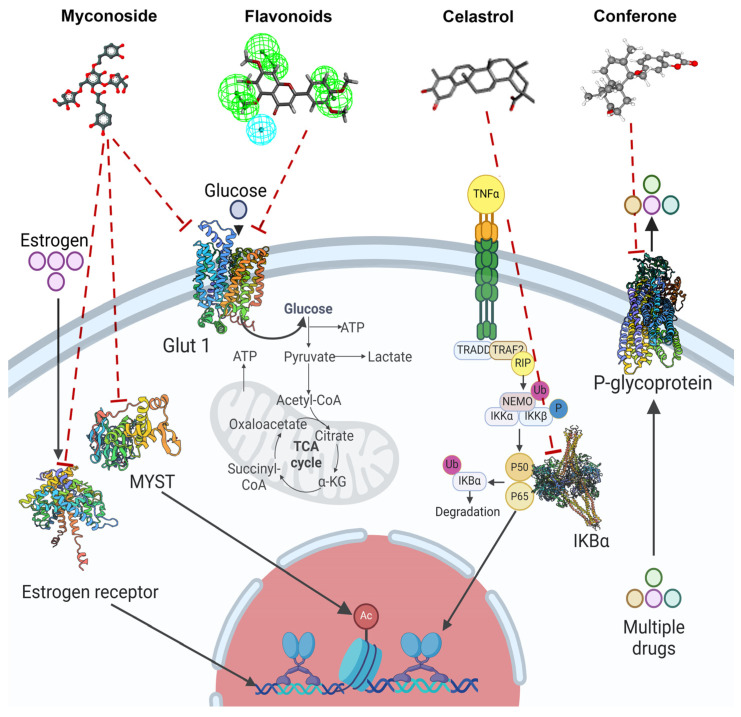
Molecular interaction mechanisms of suppression of breast cancer survival and mitigation by natural phenylpropanoid glycosides, flavonoids, terpenoids, and coumarins. Structures of compounds are represented by ball and stick diagrams with their charged groups involved in interactions. The structure of myconoside was downloaded from Japan Chemical Substance Dictionary (Nikkaji); the common feature pharmacophores of flavonoids for interaction with Glut 1 represented with green and blue spheres is according to [55]; structures of celastrol and conferone were downloaded from PubChem. Proteins of interactions are represented by ribbons downloaded from PDB illustrated in the context of the processes and pathways in breast cancer cells. Red dashed lines represent the interactions of inhibition of metabolites with target proteins.

Flavonoids belong to the group of substances containing polyphenols, where they have been described as being about 4000 and they contain 15-carbon derivatives of beta-gamma-pyrone. They are divided into different classes, such as flavanones, flavans, proanthocyanidins [7], coumarins, and coumarin-related compounds [53]. A comparative study of flavonoids from different subclasses and their effects on breast cancer cell lines established the leading role of the 2,3-double bond in the C-ring in cytotoxicity linked with mitochondrial impairment [54]. Docking analysis of 98 flavonoids with GLUT1 transporter, overexpressed in several carcinomas, has shown that the transporter is a target for flavonoids. Screening for cytotoxicity on a carcinoma cell line has shown that eight of them (apigenin, kaempferol, eupatilin, luteolin, hispidulin, isosinensetin, sinensetin, and nobiletin) reduce cell viability to 50% by inhibition of GLUT1 transporter, the critical pharmacophores of flavonoids inhibitors being 3′hydrophobic groups and hydrogen bond acceptors [55]. The molecular mechanism of glucose metabolism inhibition in tumor cells by forming the flavonoid–GLUT1 transporter complex is shown in Figure 2. The group of flavonoids like genistein, which is isolated from soya and soya products, induces cell cycle arrest in the G2/M phase in the triple-negative MDA-MB231 cell line, and the mechanism of the arrest is mediated by ERK1/2 kinase pathway activation and downregulation of Cdk1, cyclin B1, and Cdc25 C [56]. Genistein, in combination with doxorubicin, exhibited a synergistic effect on MCF-7/Adr drug-resistant cells by an increase in the intracellular accumulation of doxorubicin and inhibition of HER2/neu expression [57]. It was demonstrated that the bioflavonoid quercetin, which is characterized by low in vivo toxicity, increases the inhibitory effect of doxorubicin in MCF-7 Adr-resistant breast cancer cells [58]. Combined again with doxorubicin, quercetin induced rejection of 4T1 breast cancer in mice [59]. The natural flavonoid naringenin associated with doxorubicin synergistically suppressed the growth and migration of MCF7 cells [60].

#### Coumarins

Among flavonoids, coumarins have a very important role in breast cancer research on model cell lines. Coumarin is 1,2-benzopyrone or 2H-1-benzopyran-2-one. It can be found in various plant organs (roots, seeds, and leaves). Coumarins are widely available in plants—800 coumarin derivatives are known from about 100 plant families and 600 genera [53,61]. They have a polyphenolic structure and are colorless. The coumarins are divided into four groups—simple coumarins, composed of hydroxylated, alkoxilated, and alkylated coumarin derivatives and their glycosides; pyranocoumarins, which are structures of six-furan rings and fused with the benzene ring furanocoumarins, which comprise furane ring and coumarin fusion [62]; and pyrone-substituted coumarins. This group is divided into three subgroups: 4-hydroxycoumarin, 3-phenylcoumarin, and 3,4-benzocoumarin. Coumarins have biological effects on various diseases, including cancer. The mechanisms of their anticancer effects are related to the induction of apoptosis through the activation of a caspase cascade initiated by caspase 9, which is mediated by a decrease in antiapoptotic Bcl2 expression levels [63]. Their anticancer effects have been established on the MCF7 breast cancer cell model line. Treatment with coumarin derivatives increased P21 protein expression and arrested cells in the G0/G1 phase [64], Three synthesized coumarins derived from triphenylethylene inhibit angiogenesis of breast cancer cell lines and more precisely, compound TCH-5c changes endothelial cell cytoskeleton organization and migration of EA.hy926 endothelial cells [64]. Cellular treatment with them affects different cell signaling and kinase-dependent pathways that direct cancer cells to apoptosis or cell cycle arrest [63,64]. The coumarin group targets the key cell cycle regulator cdc25 and is a good option for targeted therapy of breast cancer [65]. Oral uptake of coumarins is effective. They are absorbed rapidly in the gastrointestinal tract and penetrate cells by passive diffusion through the lipid membrane. Coumarins are rapidly metabolized in the liver/excreted by the kidney, and only 2–6% reach the system circulation [66,67]. The toxicological studies of coumarins are very controversial. Synthetic coumarins have been shown to cause acute chronic and cancerogenic effects in mice and rats [66]. In contrast, studies on human and cynomolgus monkey liver fragments or hepatocytes have shown relative resistance to coumarin toxicity [66]. The use of standard breast anticancer drugs results in a multidrug resistance phenotype. Some of the plant coumarins suppress Pgp-mediated drug efflux in the MCF7 cell line, and thus, they are promising for overcoming multidrug resistance [68]. The mechanism of multidrug resistance suppression of conferone is presented in Figure 2.

### 3.2. Alkaloids in Breast Cancer Therapy

The search for well-characterized and purified anticancer drugs of plant origin has been very intense in recent years, and some of them are used in therapy, such as the vinca alkaloids [67]. Their mechanism for arresting the development of cancer cells is related to the blockage of the microtubule organization of the spindle pole body. They stop cell division and cancer development by binding it to the tubulin-microtubule-associated protein structure. A more stable complex between the alkaloid and tubulin-MAP is formed, and the stability is reflected in the prevention of the formation of the mitotic spindle, which results in abrogated mitosis and cell proliferation [6,7]. Taxol has the same mechanism of action. Such effects of taxol have been demonstrated for various cancer cells (breast adenocarcinoma cells) [6,8], but their application is limited by the multidrug resistance of cancer cells to taxol. Multidrug resistance to routinely used anticancer drugs is a challenge for scientists to search for new metabolic plant substances with antitumor potential and minimal side effects.

### 3.3. Polyphenols

#### 3.3.1. Curcumin

Curcumin (diferuloylmethane) has a polyphenolic structure and is a yellow powder derived from a plant extract of *Curcuma longa*. This substance is the subject of many studies related to its anticancer effects. Its effects mediate the inhibition of the transcription factor NFkB in the MCF7 breast cancer cell line [69] after reducing the expression of its target gene, such as COX2 and cyclin D, leading to apoptosis [6,7]. It can affect cells by binding to the tubulin-like taxol and thereby block mitosis [69]. It has an effect on normal cells and is therefore used in combination with other drugs. Paclitaxel and curcumin reduced cell viability of breast cancer cell lines MCF7 and MDA-MB231 through apoptosis, activation of caspases 3/7 and protein expression of nuclear NfkB transcription factor [70]. Curcumin has effects on cell culture models of various origins as an effective inhibitor of tyrosine-regulated kinase 2, a positive regulator of the 26S proteasome, which disrupts it and leads to impaired cell proliferation in the triple-negative breast cancer cell line MDA-MB468 [71]. The effect of curcumin on cell viability was favored by binding to jacalin molecules, which act as a natural ligand of Thomsen–Friedenreich (TF) tumor-associated antigen in this triple-negative cell line MDA-MB231 [72]. It blocks kinase pathways by NfKb signaling in combination with taxol in higher doses and can bind to DNA molecules, thus blocking cell cycle progression; besides, it can block angiogenesis, targeting the VEGF-VEGFR2 signaling pathway [69,70]. Other studies related to the combined treatment of curcumin with standardly used drugs on breast cancer cell lines show an improvement in the anticancer effects of the chemotherapeutics. Paclitaxel and curcumin combination induced apoptosis by the NfkB signaling pathway in comparison to single drug treatment [73]. Curcumin reverses doxorubicin multidrug resistance in MCF7 and MDA-MB231 cell lines, targeting ABCB4 by its ATPase activity [74]. Curcumin inhibits epithelial-mesenchymal transition induced by doxorubicin by targeting TGFβ and kinase signaling pathways PI3K/AKT in triple-negative breast cancer cell lines [75]. Studies on MDA-MB-231 cells reported synergistic combinations of curcumin with different oncotherapeutics, among which are cisplatin, vinorelbine, and ixabepilone, leading to cell cycle arrest and induction of apoptosis [76], potentiating the effect of curcumin on the cytotoxicity of paclitaxel against MDA-MB-435 breast cancer cells, and decreasing the incidence of breast cancer lung metastasis after curcumin and paclitaxel administration in the breast cancer xenograft model system [77]. In clinical trials with humans and rodents, curcumin was administered orally, intravenously, transdermally, intraperitoneally, and intratumorally [78]. The combined treatment of patients with standard therapeutic docetaxel and curcumin shows unchanged therapeutic outcomes compared to the group of only docetaxel treated patients [79]. Radiation therapy-induced dermatitis was reduced by oral administration of curcumin in clinical trials on women with noninflammatory breast cancer [80] Curcumin side effects related to diarrhea, headache, rash, and yellow stool in subjects who have received high doses of curcumin (500–12,000 mg) [81] and the prolonged use of curcumin (1–4 months) is related to an increase in serum alkaline phosphatase and lactate dehydrogenase [82]. Curcumin is usually metabolized in phase I and II biotransformation in the liver (phase I) and in the intestine and gut microbiota (II), the second phase being more intensive [83].

#### 3.3.2. Saponins

Indicacin is a polyphenol from the group of 3-terpenoid saponins purified from a methanol extract of *Fagonia indika*. It has an effect on breast cancer cell lines of different origins (MDA-MB-468 and MCF7) through PARP cleavage, caspase 3 activation, DNA fragmentation, and apoptosis activation [84]. *Morus alba* metabolite lectin has an antiproliferative effect on the MCF7 cell line [85]. Platycodin D from *Placticodon grandifloras* has a cytotoxic effect, activates caspases, and induces apoptosis in the MCF7 cell line [86]. The in vivo activity of platycodin D was established on mice with tumors induced by an injection of human metastatic breast cancer cells. Oral administration of platycodin D inhibited cell-induced osteolysis and blocked osteoclast formation and osteoclast-mediated bone resorption [87]. In combination with docetaxel, platycodin enhanced the antiproliferative effect in MCF7 and MDA-MB231 cell lines [88]. The side effects of platycodin D, as of the other saponins, are related to the induction of hemolytic activity [89], which could be solved by chemical modifications of their structure.

#### 3.3.3. Myconoside

The anticancer effects of the phenyl propanoid glycoside myconoside, identified in the methanol extract fraction of the resurrection plant *Haberlea rhodopensis*, were established on MCF7 and MDA-MB231 breast cancer cell lines. The cytotoxic and antiproliferative effects of the myconoside-enriched fraction have been demonstrated, and the docking analysis of myconoside with estrogen receptor, glucose transporter, and MYST acetyltransferase provides a basis for the explanation of the molecular mechanisms of their anticancer effects [90] and prospects for the future search of targeted therapy based on myconoside treatment. The molecular mechanism of the myconoside effect on the MCF7 cell line is presented in Figure 2. Docking analysis predicts that it blocks estrogen receptors, glucose transporter GLUT1, and MYST acetyltransferase and, in this way, reduces cell growth and proliferation. MDA-MB231 triple negative cell line is influenced by linking to the glucose transporter and MYST acetyltransferase [90].

### 3.4. Plant Metabolites with Anticancer Effects on Triple Negative Cell Lines

Different groups of plant metabolites have anticancer effects on triple-negative cell lines that do not express estrogen receptors, progesterone receptors, and the epidermal growth factor receptor (HER1). These characteristics make them unresponsive to hormone replacement therapy and with a higher invasive and metastatic potential [91].

#### 3.4.1. Piperine

Piperine from black pepper arrests MDA-MB231 and MDA-MB468 cells by blocking the activation of the phosphatidyl inositol 3 kinase pathway and triggering the caspase-dependent mitochondrial apoptosis pathway. Piperine-treated cells showed lower MMP2/9 expression and migration potential [92]. The effects of alkaloids such as piperlongumine (*Piper longum*), berberine, a quaternary ammonium alkaloid extracted from *Coptis chinensis*, indirubin-3-monoxime found in *Indigo naturalis*, are associated with the blocking of kinase signaling pathways and reducing the migratory potential of triple-negative breast cancer cells [93,94,95]. Piperine, as other described alkaloid agents in this paragraph, does not have side effects, and the application of piperine in combination with other conventional drugs like paclitaxel is very effective. It is absorbed by the intestinal tract and metabolized in the liver and, kidney, and excreted in bile and urine [96].

#### 3.4.2. Terpenoids

The terpenoid group is another plant metabolite group with established effects on triple-negative breast cancer cell lines. Tanshinone I and Tanshinone IIA found in the Dan Shen root of *Salvia miltiorrhiza* affect the MDA-MB231 cell line by reducing cell growth and vascular endothelial growth factor (VEGF) expression, thereby reducing the proliferation level via mTOR/p70S6K/4 E-BP1 signaling pathway [97]. Eupalinolide J (EJ), a sesquiterpene lactone found in *Eupatorium lindleyanum*, astragaloside IV, an active triterpenoid from *Radix astragali* found in the roots of *Astragalus membranaceus Bunge*, Betulinic acid, and *Dillenia suffruticosa Martelli* root extract KHF16 (24-acetylisodahurinol-3-O-D-xylopyranoside), a triterpenoid found in the rhizomes of *Cimicifuga foetida*, the pseudopterosins, a class of marine diterpene glycosides, extracted from the gorgonian sea whip *Antillogorgia elisabethae*, have anticancer effects on triple-negative breast cancer cell lines. The effects of terpenoids on triple-negative cell lines can be classified based on their molecular mechanisms leading to cell death. KHF16 (24-acetylisodachurinol-3-O-D-xylopyranoside) triggers cell cycle arrest and apoptosis in some triple-negative cell lines, promoting G2/M phase cell cycle arrest and NF-Kb pathway-mediated necrosis [98]. The terpenoids inhibit cell proliferation by multiple targets. The molecular mechanism of their anticancer activity is related to the NfkB pathway. Triterpene celastrole inhibits tumor growth of mouse xenographs of the triple-negative cell lin MDA-MB-435 e to 60% by NfKb inactivation and activates apoptotic effects induced by tumor necrosis factor α. NfkB inactivation inhibits its DNA binding capacity (Figure 2). The celastrole molecule suppresses NfkB, targeting its cysteine 179 [99]. The effects of terpenoids on triple-negative cell lines are limited in clinical trial translation because of their poor absorption and low availability, which implies the need for their structural modification in synthetic analogs. Their use in combination with standard drugs like doxorubicin, cis platina, and paclitaxel diminishes multidrug resistance of tumor cells and increases the effectiveness of chemotherapy [100].

Well-characterized polyphenols with their anticancer mechanisms clarified on cancer cell lines of different origins are described in Table 1.

## 4. Naturally Occurring Plant Bioactive Peptides and Mechanisms of Their Cytotoxic Effects on Breast Cancer Cell Lines

Other compounds of plant origin with a potential in cancer therapy are plant bioactive peptides, which are also a subject of intense research and, due to the different chemical nature compared to secondary metabolites, could have different targets and may present synergism if combined with conventional drugs or phytochemicals. Recently an increasing interest has manifested in the application of bioactive peptides in cancer therapy as an alternative or supplement to conventional drugs, especially in diminishing the severe side effects and reversing multidrug resistance [101]. Anticancer peptides are usually composed of 10–100 amino acids linked by peptide bonds in a linear or cyclic manner [102,103]. They present better biocompatibility and biodegradability compared to conventional drugs, as well as structural variability and high affinity binding, which makes them versatile tools for selective tumor targeting; however, some immunogenicity or in vivo degradability by proteases cannot be excluded [101]. Bioactive peptides could be obtained from virtually all living organisms, including more than 3000 plant species; plant families of *Fabaceae*, *Asteraceae*, *Solanaceae*, *Cucurbitaceae*, and *Brassicaceae* are particularly rich in anticancer peptides [101,102]. Based on their source, bioactive peptides can be natural (well-defined molecules or protein hydrolysates), artificially modified (for example, to resist proteolysis or to carry some cargo molecules), or in vitro synthesized. Lately, artificial intelligence has been actively used for screening possible adverse effects, immunogenicity, or peptidase degradability [102]. In the application of bioactive peptides, an advantage is taken of the different properties of cancer cells compared to normal ones, such as higher membrane fluidity with increased cell surface area and higher net negative charge and certain overexpressed receptors on tumor cell surface [104]. According to the mode of anticancer action, peptides could disrupt cell membrane integrity by forming pores, could bind to receptors and activate downstream signaling, and could penetrate the cell and exert their cytotoxic effects inside [101,102,103,104]. There are several reviews summarizing the current research on anticancer peptides, including protein hydrolysates [101,102,104,105]. It is essential to know the exact mechanism of anticancer action and to relate structure to function, which is possible only for peptides with well-known amino acid sequences. In the present review, the attention is focused on two structurally well-characterized kinds of peptides—soybean lunasin and Bowman–Birk type legume protease inhibitors, whose cytotoxic effects on breast cancer model cell lines have been thoroughly studied. Moreover, it has been established that a co-occurring protease inhibitor protects lunasin from proteolytic degradation, thus increasing its bioavailability [106].

### 4.1. Lunasin

Lunasin is a soybean-derived bioactive peptide of 5.5 kDA (43 amino acids, SKWQHQQDSCRKQLQGVNLTPC-EKHIMEKIQG-RGD-DDDDDDDD, four structural fragments). It was discovered in 1987, and its antimitotic and cell death effects were established 12 years later [107]. The RGD motif (Arg-Gly-Asp) competes with integrins, suppressing the integrin-mediated signaling pathway [108]. This motif mediates the binding of the peptide to receptors and its internalization into tumor cells; besides, binding through the RGD motif could directly activate caspase-3 and promote apoptosis. The C-terminal aspartic acid residues can interact with chromatin, and the second fragment of nine amino acids is responsible for binding to the histone core [107]. Lunasin structure presents highly disordered and flexible features typical for intrinsically disordered proteins, which participate in transcription and translation regulation, DNA condensation, cell cycle, mitosis, and apoptosis [107]. Lunasin has no inhibiting effects on the normal breast cancer cell line MCF-10A, whereas 50% inhibition of proliferation in the triple-negative MDA-MB-231 breast cancer cell line is achieved at a twice lower concentration than that of the hormone-responsive MCF-7 cell line. In both cancer cell lines, lunasin treatment decreased aromatase gene expression and activity, vascular endothelial growth factor (VEGF) secretion and cell vitality, and induced cell apoptosis [107]. Estrogen receptor (ER)α gene expression was decreased by lunasin treatment, and ERβ gene levels were significantly increased in MDA-MB-231 cells [109]. Transcriptomic and proteomic analysis of breast cancer cell line MDA-MB-231 treated with synthetic lunasin and lunasin from overexpressing transgenic maize demonstrated apoptosis activation by significant upregulation of cysteinyl aspartate specific proteinase (CASP) 3, CASP 7, and CASP 14, almost 10-fold increase in Bax/Bcl-2 ratio, and down-regulation of DNA replication genes [110,111]. In the MCF7 cell line, lunasin up-regulated tumor suppressor phosphatase and tensin homolog deleted in chromosome ten (PTEN) promoter activity, increased PTEN transcript and protein levels and enhanced nuclear PTEN localization, and the induced apoptosis was p53-independent [112]. In both MCF7 and MDA-MB-231 cell lines, 10–20 μM lunasin inhibited the expression of matrix metalloproteinase (MMP)-2/-9, the phosphorylation of focal adhesion kinase (FAK), Src, Akt, ERK and nucleus translocation of NF-κB, thus demonstrating the possibility of metastasis suppression in breast cancer cells through integrin-mediated FAK/Akt/ERK and NF-κB signaling pathways and downregulation of MMP-2/-9 [108]. The anticancer action of lunasin is preserved by a protease inhibitor Bowman–Birk type [106].

### 4.2. Protease Inhibitors

Proteases have a crucial role in maintaining proteostasis in normal cells and the extracellular matrix [113,114]. Key proteases in this respect are the ubiquitin-proteasome system in the cytoplasm, the human caseinolytic protease p (hClpP) in mitochondria, which is a serine-type protease, the cysteine protease cathepsin B of the autophagy lysosomal system, and matrix metalloprotease family which participates in extracellular matrix remodeling [113]. The 26S proteasome coordinates the regulation and degradation of redundant, unwanted, and misfolded proteins, as well as short living regulatory proteins critical for cell growth, cell cycle progress, signaling pathways, and pro-apoptotic and antiapoptotic proteins. Tumor growth, invasion and metastasis are associated with upregulation of the main proteolytic activities, which were revealed as promising drug targets. Numerous small-molecule protease inhibitors have been developed, some of them being in clinical practice or the preclinical stage; however, the therapeutic effect has been not as strong as expected based on in vitro results on cell cultures [93]. One possible reason could be the complex role of certain proteases, which could be dual—pro- and anti-autofagy [115], as well as the complex interplay between various proteases, including both up- and downregulation [114].

Other sources of therapeutic agents targeting proteolysis in tumor cells are the protease inhibitors of plant origin, such as Kunitz trypsin inhibitors (18–24 kDa), Bowman–Birk inhibitors (6–9 kDa, 60–80 amino acids), phytocystatins (10–23 kDa) recently reviewed by [116]. *Leguminosae* and *Gramineae* seeds are particularly rich in protease inhibitors [117]. Of special interest is the two-headed trypsin-chymotrypsin inhibitor from soybean, originally described by Bowman in 1946 and characterized by Birk in 1960. This inhibitor has a characteristic tightly packed molecular structure with seven disulfide bonds, providing exceptional thermo- and pH-stability, and two opposite active centers, enabling independent inhibition of two target serine proteases [116,117]. A serine-type protease activity has been identified in the cytosol of cancer cells which was inhibited by soybean Bowman–Birk protease inhibitor (BBPI) [118]. The mechanisms of the BBPI antitumor effect have been studied in detail in breast cancer cell lines. In MCF7, it was established that Bowman–Birk inhibitor specifically inhibits the proteasomal chymotrypsin-like activity and suppresses cell proliferation through the accumulation of MAP kinase phosphatase-1, accompanied by accumulation of ubiquitinated proteins and the proteasome substrates, p21Cip1/WAF1 and p27Kip1, downregulation of cyclin D1 and cyclin E and cell cycle arrest at G1/S phase, and decrease of phosphorylated extracellular signal-related kinases (ERK1/2) [119]. BBI had no inhibitory effects on EGF-stimulated activation of ERK1/2 and Akt [119]. A similar anticarcinogenic effect has been established for BBPI from *Vigna unguiculata* seeds on MCF7 cell line—cell cycle arrest in S and G2/M phase, proteasome inhibition, apoptosis and lysosome membrane permeabilization; the cytostatic effects were accompanied by alteration in nuclear morphology, plasma membrane fragmentation, cytoplasm disorganization, presence of double-membrane vesicles, mitochondrial swelling, and an increase in the size of lysosomes, DNA fragmentation, mitochondrial membrane potential reduction, and cytoplasm acidification [120]. In another study on the effects of purified BBPI from *Vigna unguiculata* seeds on cell lines MCF7, MDA MB 231, and MCF10A, a cytostatic effect at the G2/M phase of the cell cycle was found along with apoptosis induction in a caspase-dependent manner through mitochondrial impairment and oxidative damage, following proteasome 20S inhibition, with no cytotoxic effect on normal mammary epithelial cells [121]. The Bowman–Birk inhibitor affected NF-kB target gene expression in both MCF7 and MDA MB 231 breast cancer lines [121]. Soybean BBI inhibited the cell growth of the MDA-MB-231 cell line in a dose-dependent manner, with an IC50 of 200 μg/mL, altered the expression of autophagy-related genes Atg5, Beclin1, light chain 3-II, and sequestosome1 and increased the Bax/Bcl2 ratio. Thus, Bowman–Birk protease inhibitor-induced apoptosis by changing the Bax/Bcl2 expression ratio [122]. A limited number of studies have reported enhanced cytotoxicity of the combined treatment with cisplatin and BBI on MCF7 cells without significant adverse effects on normal cells [123,124]. It is interesting to note that the Bowman–Birk protease inhibitor has been reported to possess radioprotective activity, which is related to the chymotrypsin-inhibitory region of its molecule [125]. The radioprotective effect of BBPI was established for normal fibroblasts but not for the transformed fibroblasts [126].

## 5. Conclusions and Future Directions

The search for convenient breast cancer treatment has become increasingly important due to the development of multidrug resistance and the severe side effects of conventional chemotherapy [127]. In this respect, plant natural resources in terms of a variety of secondary metabolites and bioactive peptides could be of great value. Plant secondary metabolites have a wide distribution, and some of them have been used in traditional medicine for centuries. They present a broad spectrum of health promoting activities—antioxidant, antimicrobial, antidiabetic, antihypertensive, neurostabilising, etc. This fact makes them good additives to the standard anticancer treatment for the improvement of the general condition and quality of life of the patients. On the other side, they show different anticancer effects targeting various molecular receptors and, in this way, modulating signal pathways in cancer cells towards cell death. Some of these substances are lipid soluble; they pass through the membranes and target key molecules, increasing apoptosis rate or abrogating kinase signaling pathways, which are essential in breast cancer transformation. Accumulated experimental evidence supports that some plant-derived substances have the capacity to overcome multidrug resistance and in combination with standard anticancer drugs, could increase treatment effectiveness. They interfere with the complex signaling pathways in tumor development and exert cytotoxic effects by various mechanisms [128]. An added value is that these plant-derived substances differentiate nonmalignant and malignant cells, and in the majority of cases, they do not exert cytostatic/cytotoxic effects on the normal cell lines [95]. Another advantage is the possibility of inhibiting the highly aggressive triple-negative breast cancer forms, which cannot be influenced by hormonal therapy.

Disadvantages in applying plant secondary metabolites in combined cancer treatment are linked to their low bioavailability, difficulties in accumulation of therapeutic doses of some of them because of their insolubility in water, not well-known pharmacokinetic and pharmacodynamics properties, and possible toxicity at higher doses. Low bioavailability could be solved by the creation of synthetic homologs with the right modifications of their active groups or the use of different carriers targeting them to breast cancer by recognizing markers of breast cancer cells. Some plant metabolites could have side effects but data are controversial and depend on the experimental animals used. The disadvantages of applying bioactive peptides are linked to their susceptibility to protease digestion, some immunogenicity, and undesired reactivity.

Despite disadvantages, plant anticancer compounds are a valuable additional resource in cancer treatment, and ¼ to ½ of the patients diagnosed with cancer complement treatment with conventional drugs with herbs [129]. In this respect, it is important to know the molecular mechanisms of action in the active constituents and to predict the interactions with a chemotherapeutic, which could be of three kinds—synergism, an additive effect, or antagonism (if two compounds bind to the same target). Knowledge of the molecular mechanism of cytotoxic action, target sites, doses, and pharmacokinetics will be at hand to go beyond the trial-and-error approach and predict possible interactions. In this respect, the use of model cell lines with defined molecular characteristics close to those of in vivo tumor cells is of enormous importance to check the effectiveness of certain combinations; however, the experiments on cell lines cannot be extrapolated directly to cancer situation in vivo [129].

Knowledge of the exact molecular mechanisms of the observed cytotoxic effects permits combined treatments with substances targeting different pathways of cytotoxic action. Despite recent progress, a largely unexplored field is the use of drugs, which target the activated proteolytic machinery in tumor cells [113] and the combined treatment with natural substances and/or drugs with different molecular targets, which could additionally destabilize malignant cells and could be a way to reverse the development of multidrug resistance [101,114].

## Figures and Tables

**Table 1:** Plant secondary metabolites with anticancer effects on breast cancer cell lines with established molecular mechanism.

Metabolites/ Plant Origin	Chemical Structure (PubChem Database- https://pubchem.ncbi.nlm.nih.gov/ Accessed on 20 September 2024)	Established Anticancer Activity on Breast Cancer Cell Models	Established Molecular Mechanism of Anticancer Activity	References
Coumarins- About 800 different substances found in various plants (vegetables, nuts, fruits, coffee)	Essential chemical structure 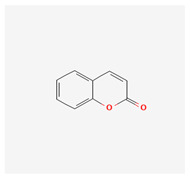	Estrogen receptor and progesterone receptor expressing MCF-7 cell line	Apoptosis activation by caspase9 pathway. Cell cycle arrest in G0/G1 phase	[63,64]
Vinca alkaloids Taxol (Paclitaxel) Derived from *Catharanthus roseus*	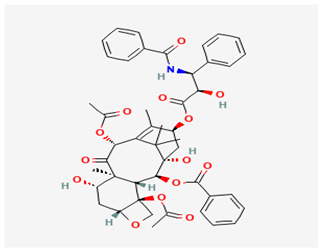	Group of substances used in standard chemotherapy that have effects on different breast cancer cell lines	Apoptosis induction, binding to DNA molecules and cell proliferation arrest, development of multidrug resistance in cells	[6,7,8]
Piperine from *Piper longum* extract	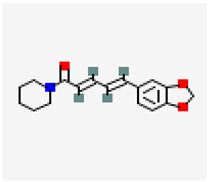	MDA-MB-231 and MDA-MB-468 triple-negative cell lines	Blocking activation of Phosphatidyl inositol 3 kinase pathway and triggering the caspase-dependent mitochondrial apoptosis	[92]
Piperlongumine from *Piper longum* extract	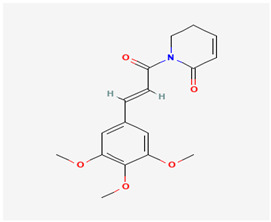	Triple-negative breast cancer cell lines	Blockage of the kinase signal pathways, decrease in migration potential	[93]
Curcumin isolated from extract of *Curcuma longa*	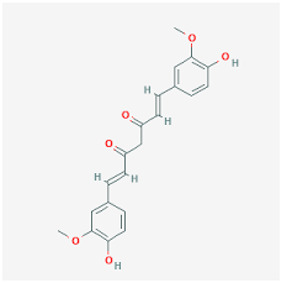	Effects on MCF-7 cell line and triple-negative cell lines like MDA-MB-231, MDA-MB-468	Apoptosis activation by inhibition of NfkB transcription factor followed by reduction of its target genes expression (*COX2*, cyclin D), Inhibition of tyrosine-regulated kinase 2, perturbation of 26S proteasome and cell cycle arrest	[6,7,68,69,70,71,72]
Myconoside * from *Haberlea rhodopensis*	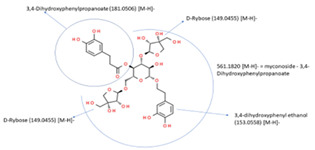	MCF-7 MDA-MB-231	Cytotoxic and antiproliferative effects	[90]
Platycodin D ** from *Placticodon grandifloras*	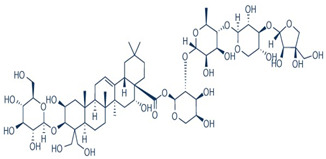	MCF-7	Cytotoxic effect activation of caspases and apoptosis	[86]
Tanshinone I Tanshinone IIA the Dan Shen root of *Salvia miltiorrhiza*	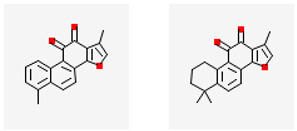	MDA-MB-231	Reduction of cell growth and VEGF expression, decrease in proliferation via mTOR/p70S6K/4 E-BP1 signaling pathway	[97]
Berberine from *Coptis chinensis*	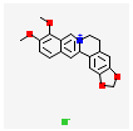	Triple-negative breast cancer cell lines	Blockage of the kinase signal pathways, decrease in migration potential	[94,95]
Eupalinolide from *Eupatorium lindleyanum*	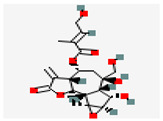	Triple-negative breast cancer cell lines	Triggering of cell cycle arrest and apoptosis	[98]
KHF16 *** From *Cimicifuga foetida*	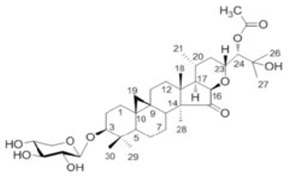	Triple-negative breast cancer cell lines MDA-MB-231, MDA-MB-468, estrogen receptor-expressing lines MCF-7 and T-47D	Cell cycle arrest in G2/M phase, NF-Kb pathway-mediated necrosis	[98]
Genistein isolated from soya and soya products	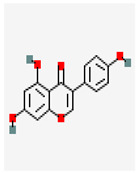	MDA-MB-231	Cell cycle arrest in G2/M phase mediated by ERK1/2 kinase pathway activation and downregulation of Cdk1, cyclin B1 and Cdc25 C	[56]

Note: The chemical structures of plant metabolites are from PubChem: https://pubchem.ncbi.nlm.nih.gov/ accessed on 20 September 2024. * The myconoside structure from *Haberlea rhodopensis* as determined by MS/MS identification in reference [90]. ** The Platycodin D structure is described following the link: https://www.selleckchem.com/products/platycodin-d.html accessed on 20 September 2024. *** KHF 16 structure is described following the [98].

## Data Availability

Data sharing is not applicable.

## References

[1] Zarei O., Benvenuti S., Ustun-Alkan F., Hamzeh-Mivehroud M., Dastmalchi S. (2016). Strategies of targeting the extracellular domain of RON tyrosine kinase receptor for cancer therapy and drug delivery. J. Cancer Res. Clin. Oncol..

[2] Plackal Adimuriyil George B., Abrahamse H. (2016). A review on novel breast cancer therapies: Photodynamic therapy and plant derived agent induced cell death mechanisms. Anti-Cancer Agents Med. Chem. (Former. Curr. Med. Chem.-Anti-Cancer Agents).

[3] https://www.iarc.who.int/news-events/current-and-future-burden-of-breast-cancer-global-statistics-for-2020-and-2040/.

[4] Avtanski D., Poretsky L. (2018). Phyto-polyphenols as potential inhibitors of breast cancer metastasis. Mol. Med..

[5] https://www.breastcancer.org/facts-statistics.

[6] Stierle A., Strobel G., Stierle D. (1993). Taxol and taxane production by Taxomyces andreanae, an endophytic fungus of Pacific yew. Science.

[7] Lindholm P. (2005). Cytotoxic Components of Plant Origin -Biological and Chemical Diversity. Ph.D. Thesis.

[8] Demain A.L., Vaishnav P. (2011). Natural products for cancer chemotherapy. Microb. Biotechnol..

[9] Mansoori B., Mohammadi A., Davudian S., Shirjang S., Baradaran B. (2017). The different mechanisms of cancer drug resistance: A brief review. Adv. Pharm. Bull..

[10] Mazumder K., Biswas B., Raja I.M., Fukase K. (2020). A review of cytotoxic plants of the Indian subcontinent and a broad-spectrum analysis of their bioactive compounds. Molecules.

[11] Kumar V., Green S., Stack G., Berry M., Jin J.R., Chambon P. (1987). Functional domains of the human estrogen receptor. Cell.

[12] Osborne C.K., Schiff R., Fuqua S.A., Shou J. (2001). Estrogen receptor: Current understanding of its activation and modulation. Clin. Cancer Res..

[13] Renoir J.M., Marsaud V., Lazennec G. (2013). Estrogen receptor signaling as a target for novel breast cancer therapeutics. Biochem. Pharmacol..

[14] Cheskis B.J., Greger J.G., Nagpal S., Freedman L.P. (2007). Signaling by estrogens. J. Cell. Physiol..

[15] Sever R., Brugge J.S. (2015). Signal transduction in cancer. Cold Spring Harb. Perspect. Med..

[16] Miziak P., Baran M., Błaszczak E., Przybyszewska-Podstawka A., Kałafut J., Smok-Kalwat J., Dmoszy ´nska-Graniczka M., Kiełbus M., Stepulak A. (2023). Estrogen Receptor Signaling in Breast Cancer. Cancers.

[17] Clusan L., Ferrière F., Flouriot G., Pakdel F. (2023). A Basic Review on Estrogen Receptor Signaling Pathways in Breast Cancer. Int. J. Mol. Sci..

[18] Bjornstrom L., Sjoberg M. (2005). Mechanisms of estrogen receptor signaling: Convergence of genomic and nongenomic actions on target genes. Mol. Endocrinol..

[19] Saha Roy S., Vadlamudi R.K. (2012). Role of estrogen receptor signaling in breast cancer metastasis. Int. J. Breast Cancer.

[20] Roskoski R. (2014). The ErbB/HER family of protein-tyrosine kinases and cancer. Pharmacol. Res..

[21] Arteaga C.L., Engelman J.A. (2014). ERBB receptors: From oncogene discovery to basic science to mechanism-based cancer therapeutics. Cancer Cell.

[22] Wee P., Wang Z. (2017). Epidermal growth factor receptor cell proliferation signaling pathways. Cancers.

[23] Wieduwilt M.J., Moasser M.M. (2008). The epidermal growth factor receptor family: Biology driving targeted therapeutics. Cell. Mol. Life Sci..

[24] Garrett T.P., McKern N.M., Lou M., Elleman T.C., Adams T.E., Lovrecz G.O., Kofler M., Jorissen R.N., Nice E.C., Burgess A.W. (2003). The crystal structure of a truncated ErbB2 ectodomain reveals an active conformation, poised to interact with other ErbB receptors. Mol. Cell.

[25] Graus-Porta D., Beerli R.R., Daly J.M., Hynes N.E. (1997). ErbB-2, the preferred heterodimerization partner of all ErbB receptors, is a mediator of lateral signaling. EMBO J..

[26] Burgess A.W. (2008). EGFR family: Structure physiology signalling and therapeutic targets. Growth Factors.

[27] Mayer I.A., Arteaga C.L. (2016). The pi3K/AKT pathway as a target for cancer treatment. Annu. Rev. Med..

[28] Ono M., Kuwano M. (2006). Molecular mechanisms of epidermal growth factor receptor (EGFR) activation and response to gefitinib and other EGFR-targeting drugs. Clin. Cancer Res..

[29] Yousefnia S., Seyed Forootan F., Seyed Forootan S., Nasr Esfahani M.H., Gure A.O., Ghaedi K. (2020). Mechanistic pathways of malignancy in breast cancer stem cells. Front. Oncol..

[30] Hosseini H., Obradović M.M., Hoffmann M., Harper K.L., Sosa M.S., Werner-Klein M., Nanduri L.K., Werno C., Ehrl C., Maneck M. (2016). Early dissemination seeds metastasis in breast cancer. Nature.

[31] Feng Y., Spezia M., Huang S., Yuan C., Zeng Z., Zhang L., Ji X., Liu W., Huang B., Luo W. (2018). Breast cancer development and progression: Risk factors, cancer stem cells, signaling pathways, genomics, and molecular pathogenesis. Genes Dis..

[32] Yang K., Wang X., Zhang H., Wang Z., Nan G., Li Y., Zhang F., Mohammed M.K., Haydon R.C., Luu H.H. (2016). The evolving roles of canonical WNT signaling in stem cells and tumorigenesis: Implications in targeted cancer therapies. Lab. Investig..

[33] Mohammed M.K., Shao C., Wang J., Wei Q., Wang X., Collier Z., Tang S., Liu H., Zhang F., Huang J. (2016). Wnt/beta-catenin signaling plays an ever-expanding role in stem cell self-renewal, tumorigenesis and cancer chemoresistance. Genes Dis..

[34] Clevers H., Nusse R. (2012). Wnt/beta-catenin signaling and disease. Cell.

[35] Nusse R., Clevers H. (2017). Wnt/beta-Catenin signaling, disease, and emerging therapeutic modalities. Cell.

[36] Ugolini F., Adélaïde J., Charafe-Jauffret E., Nguyen C., Jacquemier J., Jordan B., Birnbaum D., Pébusque M.J. (1999). Differential expression assay of chromosome arm 8p genes identifies Frizzled-related (FRP1/FRZB) and Fibroblast Growth Factor Receptor 1 (FGFR1) as candidate breast cancer genes. Oncogene.

[37] Veeck J., Geisler C., Noetzel E., Alkaya S., Hartmann A., Knüchel R., Dahl E. (2008). Epigenetic inactivation of the secreted frizzled-related protein-5 (SFRP5) gene in human breast cancer is associated with unfavorable prognosis. Carcinogenesis.

[38] Khramtsov A.I., Khramtsova G.F., Tretiakova M., Huo D., Olopade O.I., Goss K.H. (2010). Wnt/beta-catenin pathway activation is enriched in basal-like breast cancers and predicts poor outcome. Am. J. Pathol..

[39] Lin S.Y., Xia W., Wang J.C., Kwong K.Y., Spohn B., Wen Y., Pestell R.G., Hung M.C. (2000). Beta-catenin, a novel prognosticmarker for breast cancer: Its roles in cyclin D1 expression and cancer progression. Proc. Natl. Acad. Sci. USA.

[40] Schade B., Lesurf R., Sanguin-Gendreau V., Bui T., Deblois G., O’Toole S.A., Millar E.K., Zardawi S.J., Lopez-Knowles E., Sutherland R.L. (2013). beta-Catenin signaling is a critical event in ErbB2-mediated mammary tumor progression. Cancer Res..

[41] Nurse P.M. (2002). Nobel Lecture. Cyclin dependent kinases and cell cycle control. Biosci. Rep..

[42] Sutherland R.L., Musgrove E.A. (2004). Cyclins and breast cancer. J. Mammary Gland. Biol. Neoplasia.

[43] Radtke F., Raj K. (2003). The role of Notch in tumorigenesis: Oncogene or tumour suppressor?. Nat. Rev. Cancer.

[44] Reedijk M., Odorcic S., Chang L., Zhang H., Miller N., McCready D.R., Lockwood G., Egan S.E. (2005). High-level coexpression of JAG1 and NOTCH1 is observed in human breast cancer and is associated with poor overall survival. Cancer Res..

[45] Dickson B.C., Mulligan A.M., Zhang H., Lockwood G., O’Malley F.P., Egan S.E., Reedijk M. (2007). High-level JAG1mRNA and protein predict poor outcome in breast cancer. Mod. Pathol..

[46] García-Zaragoza E., Pérez-Tavarez R., Ballester A., Lafarga V., Jiménez-Reinoso A., Ramírez Á., Murillas R., Gallego M.I. (2012). Intraepithelial paracrine Hedgehog signaling induces the expansion of ciliated cells that express diverse progenitor cell markers in the basal epithelium of the mouse mammary gland. Dev. Biol..

[47] Fiaschi M., Rozell B., Bergstrom A., Toftgard R., Kleman M.I. (2007). Targeted expression of GLI1 in the mammary gland disrupts pregnancy-induced maturation and causes lactation failure. J. Biol. Chem..

[48] Miah S., Martin A., Lukong K.E. (2012). Constitutive activation of breasttumor kinase accelerates cell migration and tumor growth in vivo. Oncogenesis.

[49] Koren S., Reavie L., Couto J.P., De Silva D., Stadler M.B., Roloff T., Britschgi A., Eichlisberger T., Kohler H., Aina O. (2015). PIK3CA(H1047R) induces multipotency and multi-lineage mammary tumours. Nature.

[50] Kim E.K., Kim H.A., Koh J.S., Kim M.S., Kim K.I., Lee J.I., Moon N.M., Ko E., Noh W.C. (2011). Phosphorylated S6K1 is a possible marker for endocrine therapy resistance in hormone receptor-positive breast cancer. Breast Cancer Res. Treat..

[51] Yang Y., Leonard M., Zhang Y., Zhao D., Mahmoud C., Khan S., Wang J., Lower E.E., Zhang X. (2018). HER2-Driven breast tumorigenesis relies upon interactions of the estrogen receptor with coactivator MED1. Cancer Res..

[52] Shrihastini V., Muthuramalingam P., Adarshan S., Sujitha M., Chen J.T., Shin H., Ramesh M. (2021). Plant derived bioactive compounds, their anti-cancer effects and in silico approaches as an alternative target treatment strategy for breast cancer: An updated overview. Cancers.

[53] Seca A.M., Pinto D.C. (2018). Plant secondary metabolites as anticancer agents: Successes in clinical trials and therapeutic application. Int. J. Mol. Sci..

[54] Yadegarynia S., Pham A., Ng A., Nguyen D., Lialiutska T., Bortolazzo A., Sivryuk V., Bremer M., White J.B. (2012). Profiling flavonoid cytotoxicity in human breast cancer cell lines: Determination of structure-function relationships. Nat. Prod. Commun..

[55] Sun Y., Duan X., Wang F., Tan H., Hu J., Bai W., Wang X., Wang B., Hu J. (2023). Inhibitory Effects of Flavonoids on Glucose Transporter 1 (GLUT1): From Library Screening to Biological Evaluation to Structure-Activity Relationship. Toxicology.

[56] Li Z., Li J., Mo B., Hu C., Liu H., Qi H., Wang X., Xu J. (2008). Genistein induces G2/M cell cycle arrest via stable activation of ERK1/2 pathway in MDA-MB-231 breast cancer cells. Cell Biol. Toxicol..

[57] Xue J.P., Wang G., Zhao Z.B., Wang Q., Shi Y. (2014). Synergistic cytotoxic effect of genistein and doxorubicin on drug-resistant human breast cancer MCF-7/Adr cells. Oncol. Rep..

[58] Scambia G., Ranelletti F.O., Panici P.B., De Vincenzo R., Bonanno G., Ferrandina G., Piantelli M., Bussa S., Rumi C., Cianfriglia M. (1994). Quercetin potentiates the effect of adriamycin in a multidrug resistant MCF-7 human breast-cancer cell line: P-glycoprotein as a possible target. Cancer Chemother. Pharmacol..

[59] Du G., Lin H., Yang Y., Zhang S., Wu X., Wang M., Ji L., Lu L., Yu L., Han G. (2010). Dietary quercetin combining intratumoral doxorubicin injection synergistically induces rejection of established breast cancer in mice. Int. Immunopharmacol..

[60] Effat H., Abosharaf H.A., Radwan A.M. (2024). Combined effects of naringin and doxorubicin on the JAK/STAT signaling pathway reduce the development and spread of breast cancer cells. Sci. Rep..

[61] Küpeli Akkol E., Genç Y., Karpuz B., Sobarzo-Sánchez E., Capasso R. (2020). Coumarins and coumarin-related compounds in pharmacotherapy of cancer. Cancers.

[62] Lake B. (1999). Synthesis & pharmacological investigation of 4-hydroxy coumarin derivatives & shown as anti-coagulant. Food Chem. Tox..

[63] Lacy A., O’Kennedy R. (2004). Studies on coumarins and coumarin-related compounds to determine their therapeutic role in the treatment of cancer. Curr. Pharm. Des..

[64] Cui N., Lin D.D., Shen Y., Shi J.G., Wang B., Zhao M.Z., Zheng L., Chen H., Shi J.H. (2019). Triphenylethylene-coumarin hybrid TCH-5c suppresses tumorigenic progression in breast cancer mainly through the inhibition of angiogenesis. Anti-Cancer Agents Med. Chem..

[65] Valente S., Bana E., Viry E., Bagrel D., Kirsch G. (2010). Synthesis and biological evaluation of novel coumarin-based inhibitors of Cdc25 phosphatases. Bioorg. Med. Chem. Lett..

[66] Lake B. (1999). Coumarin Metabolism, Toxicity and Carcinogenicity: Relevance for Human Risk Assessment. Food Chem. Tox..

[67] Bogan D., Deasy B., O’Kennedy R., Smyth M. (1995). Determination of Free and Total 7-hydroxycoumarin in Urine and Serum by Capillary Electrophoresis. J. Chromatogr. B.

[68] Singh A., Singh K., Kaur K., Singh A., Sharma A., Kaur K., Kaur J., Kaur G., Kaur U., Kaur H. (2024). Coumarin as an Elite Scaffold in Anti-Breast Cancer Drug Development: Design Strategies, Mechanistic Insights, and Structure–Activity Relationships. Biomedicines.

[69] Liu J.L., Pan Y.Y., Chen O., Luan Y., Xue X., Zhao J.J., Liu L., Jia H.Y. (2017). Curcumin inhibits MCF-7 cells by modulating the NF-κB signaling pathway. Oncol. Lett..

[70] Cianfruglia L., Minnelli C., Laudadio E., Scirè A., Armeni T. (2019). Side effects of curcumin: Epigenetic and antiproliferative implications for normal dermal fibroblast and breast cancer cells. Antioxidants.

[71] Calaf G.M., Ponce-Cusi R., Carrión F. (2018). Curcumin and paclitaxel induce cell death in breast cancer cell lines. Oncol. Rep..

[72] Banerjee S., Ji C., Mayfield J.E., Goel A., Xiao J., Dixon J.E., Guo X. (2018). Ancient drug curcumin impedes 26S proteasome activity by direct inhibition of dual-specificity tyrosine-regulated kinase 2. Proc. Natl. Acad. Sci. USA.

[73] Petrova L., Gergov N., Stoup M., Zapryanova S., Van Damme E.J., Lebègue N., Liberelle M., Zasheva D., Bogoeva V. (2023). Jacalin-Curcumin Complex Sensitizes the Breast Cancer MDA-MB-231 Cell Line. Int. J. Mol. Sci..

[74] Wen C., Fu L., Huang J., Dai Y., Wang B., Xu G., Wu L., Zhou H. (2019). Curcumin Reverses Doxorubicin Resistance via Inhibition the Efflux Function of ABCB4 in Doxorubicin-resistant Breast Cancer Cells. Mol. Med. Rep..

[75] Chen W.-C., Lai Y.-A., Lin Y.-C., Ma J.-W., Huang L.-F., Yang N.-S., Ho C.-T., Kuo S.-C., Way T.-D. (2013). Curcumin Suppresses Doxorubicin-Induced Epithelial–Mesenchymal Transition via the Inhibition of TGF-β and PI3K/AKT Signaling Pathways in Triple-Negative Breast Cancer Cells. J. Agric. Food Chem..

[76] Ke C.S., Liu H.S., Yen C.H., Huang G.C., Cheng H.C., Huang C.Y., Su C.L. (2014). Curcumin-induced aurora-a suppression not only causes mitotic defect and cell cycle arrest but also alters chemosensitivity to anticancer drugs. J. Nutr. Biochem..

[77] Aggarwal B.B., Shishodia S., Takada Y., Banerjee S., Newman R.A., Bueso-Ramos C.E., Price J.E. (2005). Curcumin suppresses the paclitaxel-induced nuclear factor-kappab pathway in breast cancer cells and inhibits lung metastasis of human breast cancer in nude mice. Clin. Cancer Res..

[78] Mayo B., Penroz S., Torres K., Simón L. (2024). Curcumin Administration Routes in Breast Cancer Treatment. Int. J. Mol. Sci..

[79] Bayet-Robert M., Kwiatowski F., Leheurteur M., Gachon F., Planchat E., Abrial C., Mouret-Reynier M.-A., Durando X., Barthomeuf C., Chollet P. (2010). Phase I Dose Escalation Trial of Docetaxel plus Curcumin in Patients with Advanced and Metastatic Breast Cancer. Cancer Biol. Ther..

[80] Ryan J.L., Heckler C.E., Ling M., Katz A., Williams J.P., Pentland A.P., Morrow G.R. (2013). Curcumin for Radiation Dermatitis: A Randomized, Double-Blind, Placebo-Controlled Clinical Trial of Thirty Breast Cancer Patients. Radiat. Res..

[81] Lao C.D., Ruffin M.T., Normolle D., Heath D.D., Murray S.I., Bailey J.M., Boggs M.E., Crowell J., Rock C.L., Brenner D.E. (2006). Dose escalation of a curcuminoid formulation. BMC Complement. Altern. Med..

[82] Hewlings S.J., Kalman D.S. (2017). Curcumin: A Review of Its’ Effects on Human Health. Foods.

[83] Luca S.V., Macovei I., Bujor A., Miron A., Skalicka-Woźniak K., Aprotosoaie A.C., Trifan A. (2019). Bioactivity of dietary polyphenols: The role of metabolites. Crit. Rev. Food Sci. Nutr..

[84] Waheed A., Barker J., Barton S.J., Owen C.P., Ahmed S., Carew M.A. (2012). A novel steroidal saponin glycoside from Fagonia indica induces cell-selective apoptosis or necrosis in cancer cells. Eur. J. Pharm. Sci..

[85] Deepa M., Priya S. (2012). Purification and characterization of a novel anti-proliferative lectin from *Morus alba* L. leaves. Protein Pept. Lett..

[86] Yu J.S., Kim A.K. (2010). Platycodin D induces apoptosis in MCF-7 human breast cancer cells. J. Med. Food.

[87] Lee S.K., Park K.K., Kim H.J., Kim K.R., Kang E.J., Kim Y.L., Yoon H., Kim Y.S., Chung W.Y. (2015). Platycodin D Blocks Breast Cancer-Induced Bone Destruction by Inhibiting Osteoclastogenesis and the Growth of Breast Cancer Cells. Cell Physiol. Biochem..

[88] Tang Z.H., Li T., Gao H.W., Sun W., Chen X.P., Wang Y.T., Lu J.J. (2014). Platycodin D from Platycodonis Radix enhances the anti-proliferative effects of doxorubicin on breast cancer MCF-7 and MDA-MB-231 cells. Chin. Med..

[89] Sarikahya N.B., Nalbantsoy A., Top H., Gokturk R.S., Sumbul H., Kirmizigul S. (2018). Immunomodulatory, hemolytic and cytotoxic activity potentials of triterpenoid saponins from eight Cephalaria species. Phytomedicine.

[90] Zasheva D., Mladenov P., Rusanov K., Simova S., Zapryanova S., Simova-Stoilova L., Moyankova D., Djilianov D. (2023). Fractions of methanol extracts from the Resurrection plant Haberlea rhodopensis have anti-breast cancer effects in model cell systems. Separations.

[91] Thilagavathi R., Priyankha S., Kannan M., Prakash M., Selvam C. (2023). Compounds from diverse natural origin against triple-negative breast cancer: A comprehensive review. Chem. Biol. Drug Des..

[92] Greenshields A.L., Doucette C.D., Sutton K.M., Madera L., Annan H., Yaffe P.B., Knickle A.F., Dong Z., Hoskin D.W. (2015). Piperine inhibits the growth and motility of triple-negative breast cancer cells. Cancer Lett..

[93] Delaney L.M., Farias N., Ghassemi Rad J., Fernando W., Annan H., Hoskin D.W. (2020). The natural alkaloid Piperlongumine inhibits metastatic activity and epithelial-to-Mesenchymal transition of triple-negative mammary carcinoma cells. Nutr. Cancer.

[94] Zhai H.Y., Zhao C., Zhang N., Jin M.N., Tang S.A., Qin N., Kong D.X., Duan H.Q. (2012). Alkaloids from Pachysandra terminalis inhibit breast cancer invasion and have potential for development as antimetastasis therapeutic agents. J. Nat. Prod..

[95] Ali Abdalla Y.O., Subramaniam B., Nyamathulla S., Shamsuddin N., Arshad N.M., Mun K.S., Awang K., Nagoor N.H. (2022). Natural products for cancer therapy: A review of their mechanism of actions and toxicity in the past decade. J. Trop. Med..

[96] Quijia C.R., Chorilli M. (2022). Piperine for treating breast cancer: A review of molecular mechanisms, combination with anticancer drugs, and nanosystems. Phytother. Res..

[97] Li G., Shan C., Liu L., Zhou T., Zhou J., Hu X., Chen Y., Cui H., Gao N. (2015). Tanshinone IIA inhibits HIF-1α and VEGF expression in breast cancer cells via mTOR/p70S6K/RPS6/4E-BP1 signaling pathway. PLoS ONE.

[98] Kong Y., Chen C. (2015). KHF16, a new cycloartane triterpenoid isolated from Cimicifuga foetida, suppresses triple-negative breast cancer by inhibiting the NF-κB signaling pathway. Cancer Res..

[99] Yang H., Ping Dou Q. (2010). Targeting apoptosis pathway with natural terpenoids: Implications for treatment of breast and prostate cancer. Curr. Drug Targets.

[100] Kamran S., Sinniah A., Abdulghani M.A., Alshawsh M.A. (2022). Therapeutic Potential of Certain Terpenoids as Anticancer Agents: A Scoping Review. Cancers.

[101] Huang Y., Peng H., Zeng A., Song L. (2023). The role of peptides in reversing chemoresistance of breast cancer: Current facts and future prospects. Front. Pharmacol..

[102] Ghaly G., Tallima H., Dabbish E., Badr ElDin N., Abd El-Rahman M.K., Ibrahim M.A.A., Shoeib T. (2023). Anti-Cancer Peptides: Status and Future Prospects. Molecules.

[103] Fang X.Y., Chen W., Fan J.T., Song R., Wang L., Gu Y.H., Zeng G.Z., Shen Y., Wu X.F., Tan N.H. (2013). Plant cyclopeptide RA-V kills human breast cancer cells by inducing mitochondria-mediated apoptosis through blocking PDK1–AKT interaction. Toxicol. Appl. Pharmacol..

[104] Chiangjong W., Chutipongtanate S., Hongeng S. (2020). Anticancer peptide: Physicochemical property, functional aspect and trend in clinical application. Int. J. Oncol..

[105] Li Q.Z., Zhou Z.R., Hu C.Y., Li X.B., Chang Y.Z., Liu Y., Wang Y.L., Zhou X.W. (2023). Recent advances of bioactive proteins/polypeptides in the treatment of breast cancer. Food Sci. Biotechnol..

[106] Hsieh C.C., Hernández-Ledesma B., Jeong H.J., Park J.H., de Lumen B.O. (2010). Complementary roles in cancer prevention: Protease inhibitor makes the cancer preventive peptide lunasin bioavailable. PLoS ONE.

[107] Kaufman-Szymczyk A., Kaczmarek W., Fabianowska-Majewska K., Lubecka-Gajewska K. (2023). Lunasin and Its Epigenetic Impact in Cancer Chemoprevention. Int. J. Mol. Sci..

[108] Jiang Q., Pan Y., Cheng Y., Li H., Liu D., Li H. (2016). Lunasin suppresses the migration and invasion of breast cancer cells by inhibiting matrix metalloproteinase-2/-9 via the FAK/Akt/ERK and NF-κB signaling pathways. Oncol. Rep..

[109] Hsieh C.C., Wu C.H., Peng S.H., Chang C.H. (2023). Seed-derived peptide lunasin suppressed breast cancer cell growth by regulating inflammatory mediators, aromatase, and estrogen receptors. Food Nutr. Res..

[110] Hao Y., Guo H., Hong Y., Fan X., Su Y., Yang X., Ren G. (2022). Lunasin peptide promotes lysosome-mitochondrial mediated apoptosis and mitotic termination in MDA-MB-231 cells. Food Sci. Hum. Wellness.

[111] Hao Y., Wu B., Li M., Yuan M., Qiao L., Zhao J., Zheng X., Li X., Wang Y., Wang Y. (2024). Functional exploration of lunasin peptide in transgenic maize (*Zea mays* L.) and its role in controlling mitophagy in MDA-MB-231 cells. Food Biosci..

[112] Pabona J.M.P., Dave B., Su Y., Montales M.T.E., De Lumen B.O., De Mejia E.G., Rahal O.M., Simmen R.C. (2013). The soybean peptide lunasin promotes apoptosis of mammary epithelial cells via induction of tumor suppressor PTEN: Similarities and distinct actions from soy isoflavone genistein. Genes Nutr..

[113] Song R., Qiao W., He J., Huang J., Luo Y., Yang T. (2021). Proteases and their modulators in cancer therapy: Challenges and opportunities. J. Med. Chem..

[114] Hoelzen L., Mitschke J., Schoenichen C., Hess M.E., Ehrenfeld S., Boerries M., Miething C., Brummer T., Reinheckel T. (2022). RNA Interference screens discover proteases as synthetic lethal partners of PI3K inhibition in breast cancer cells. Theranostics.

[115] Hashemi M., Paskeh M.D.A., Orouei S., Abbasi P., Khorrami R., Dehghanpour A., Esmaeili N., Ghahremanzade A., Zandieh M.A., Peymani M. (2023). Towards dual function of autophagy in breast cancer: A potent regulator of tumor progression and therapy response. Biomed. Pharmacother..

[116] Cid-Gallegos M.S., Corzo-Ríos L.J., Jiménez-Martínez C., Sánchez-Chino X.M. (2022). Protease inhibitors from plants as therapeutic agents—A review. Plant Foods Hum. Nutr..

[117] Gitlin-Domagalska A., Maciejewska A., Dębowski D. (2020). Bowman-Birk inhibitors: Insights into family of multifunctional proteins and peptides with potential therapeutical applications. Pharmaceuticals.

[118] Moy L.Y., Billings P.C. (1994). A proteolytic activity in a human breast cancer cell line which is inhibited by the anticarcinogenic Bowman-Birk protease inhibitor. Cancer Lett..

[119] Chen Y.W., Huang S.C., Lin-Shiau S.Y., Lin J.K. (2005). Bowman–Birk inhibitor abates proteasome function and suppresses the proliferation of MCF7 breast cancer cells through accumulation of MAP kinase phosphatase-1. Carcinogenesis.

[120] Joanitti G.A., Azevedo R.B., Freitas S.M. (2010). Apoptosis and lysosome membrane permeabilization induction on breast cancer cells by an anticarcinogenic Bowman–Birk protease inhibitor from Vigna unguiculata seeds. Cancer Lett..

[121] Mehdad A., Brumana G., Souza A.A., Barbosa JA R.G., Ventura M.M., De Freitas S.M. (2016). A Bowman–Birk inhibitor induces apoptosis in human breast adenocarcinoma through mitochondrial impairment and oxidative damage following proteasome 20S inhibition. Cell Death Discov..

[122] Kyani S., Akrami H., Mostafaei A., Akbari S., Salehi Z. (2021). Inhibitory effect of Bowman–Birk protease inhibitor on autophagy in MDAMB231 breast cancer cell line. J. Cancer Res. Ther..

[123] Zhang L., Wan X.S., Donahue J.J., Ware J.H., Kennedy A.R. (1999). Effects of the Bowman-Birk Inhibitor on Clonogenic Survival and Cisplatin- or Radiation-Induced Cytotoxicity in Human Breast, Cervical, and Head and Neck Cancer Cells. Nutr. Cancer.

[124] Lanza A., Tava A., Catalano M., Ragona L., Singuaroli I., della Cuna FS R., della Cuna G.R. (2004). Effects of the Medicago scutellata trypsin inhibitor (MsTI) on cisplatin-induced cytotoxicity in human breast and cervical cancer cells. Anticancer Res..

[125] Gueven N., Dittmann K., Mayer C., Rodemann H.P. (1998). The radioprotective potential of the Bowman–Birk protease inhibitor is independent of its secondary structure. Cancer Lett..

[126] Dittmann K., Löffler H., Bamberg M., Rodemann H.P. (1995). Bowman–Birk proteinase inhibitor (BBI) modulates radiosensitivity and radiation induced differentiation of human fibroblasts in culture. Radiother. Oncol..

[127] Lozon L., Ramadan W.S., Kawaf R.R., Al-Shihabi A.M., El-Awady R. (2024). Decoding cell death signalling: Impact on the response of breast cancer cells to approved therapies. Life Sci..

[128] Naeem M., Iqbal M.O., Khan H., Ahmed M.M., Farooq M., Aadil M.M., Jamaludin M.I., Hazafa A., Tsai W.-C. (2022). A Review of Twenty Years of Research on the Regulation of Signaling Pathways by Natural Products in Breast Cancer. Molecules.

[129] HemaIswarya S., Doble M. (2006). Potential synergism of natural products in the treatment of cancer. Phytother. Res. An. Int. J. Devoted Pharmacol. Toxicol. Eval. Nat. Prod. Deriv..

